# Differences of Urinary Arsenic Metabolites and Methylation Capacity between Individuals with and without Skin Lesions in Inner Mongolia, Northern China

**DOI:** 10.3390/ijerph110707319

**Published:** 2014-07-18

**Authors:** Qiang Zhang, Yongfang Li, Juan Liu, Da Wang, Quanmei Zheng, Guifan Sun

**Affiliations:** 1 Department of Occupational and Environmental Health, School of Public Health, Tianjin Medical University, No. 22, Qi Xiang Tai Road, Heping District, Tianjin 300070, China; E-Mail: qiangzhang@tijmu.edu.cn; 2 Environment and Non-Communicable Disease Research Center, School of Public Health, China Medical University, No. 92, Bei Er Road, Heping District, Shenyang 110001, China; E-Mails: liyongfang_17@163.com (Y.L.); 472594283@163.com (D.W.); qmzheng@mail.cmu.edu.cn (Q.Z.); 3 Library of Tianjin Medical University, Tianjin Medical University, No. 22, Qi Xiang Tai Road, Heping District, Tianjin 300070, China; E-Mail: libliujuan@tijmu.edu.cn

**Keywords:** arsenic, groundwater, skin lesion, methylation capacity, metabolism

## Abstract

Incomplete arsenic (As) methylation has been considered a risk factor of As-related diseases. This study aimed to examine the difference of urinary As metabolites and the methylation capacity between subjects with and without skin lesions. Urinary inorganic arsenic (iAs), monomethylarsonic acid (MMA), and dimethylarsinic acid (DMA) were analyzed. The percentage of each As species (iAs%, MMA%, and DMA%), the primary methylation index (PMI) and secondary methylation index (SMI) were calculated. The results showed that subjects with skin lesions have higher levels of urinary iAs (99.08 *vs.* 70.63 μg/g Cr, *p* = 0.006) and MMA (69.34 *vs.* 42.85 μg/g Cr, *p* = 0.016) than subjects without skin lesions after adjustment for several confounders. Significant differences of urianry MMA% (15.49 *vs.* 12.11, *p* = 0.036) and SMI (0.74 *vs.* 0.81, *p* = 0.025) were found between the two groups. The findings of the present study suggest that subjects with skin lesions may have a lower As methylation capacity than subjects without skin lesions.

## 1. Introduction

Arsenic (As) exists ubiquitously in the environment. Globally, an estimated 130 million people expose to As from drinking water [1]. Groundwater As contamination in China remains a serious public health problem. A recent study reported that 19.6 million people in China could be exposed to As-contaminated drinking water (As level above 10 µg/L) [2]. It is well established that chronic As exposure is associated with a variety of health hazards in a dose-response pattern, including skin lesions [3,4]. However, recent studies indicated that the development of As-related diseases is not only determined by the levels of As exposure, but also by the variation of As metabolism [5,6,7].

In drinking water, inorganic As (iAs) is usually found in the form of arsenate (As^V^) and arsenite (As^III^). The ingested iAs is mainly metabolized in the human liver and two pathways concerning As metabolism are proposed. The generally accepted classical metabolic pathway consists of a series of reduction and oxidative methylation, which is catalyzed by reductases and methyltransferases [8]. In the reactions, the pentavalent oxidative As species is formed before the respective trivalent As species as fellows: iAs^V^ → iAs^III^ → monomethylarsonic acid (MMA^V^) → monomethylarsonous acid (MMA^III^) → dimethylarsinic acid (DMA^V^) → dimethylarsinous acid (DMA^III^). The newly proposed pathway suggests that trivalent As species are formed before pentavalent species and MMA^V^ and DMA^V^ are proposed as the end products during As metabolism [9]. Although there is difference, both pathways indicate that methylation plays a key role in understanding As biotransformation. In general, trivalent arsenicals are more toxic than their pentavalent analogs [10]. Recent studies indicated that MMA^III^ is the most toxic intermediate during As metabolism [11]. Arsenic methylation process is incomplete and iAs along with MMA and DMA are excreted in human urine. Therefore, the percentages of urinary As metabolites (iAs%, MMA%, DMA%) can give a reflection of the As methylation efficiency in human body. In addition, primary methylation index (PMI) and secondary methylation index (SMI) are usually used as indicators for evaluating the methylation capacity of individuals.

Higher MMA% value and lower DMA% and SMI values suggest an incomplete methylation of As in human body, which probably indicates higher MMA^III^ at cellular level. Epidemiologic studies have shown that reduced As methylation capacity is associated with As-related diseases including skin lesions, skin cancer, bladder cancer and hypertension [12,13,14,15]. The variability in As methylation capacity can be partially explained by genetic polymorphisms [6,16,17]. However, a number of studies showed that demographic characteristics and environmental factors such as gender, age, body mass index (BMI), personal lifestyle, as well as dietary factors and nutritional status can influence the As metabolism [18,19,20]. Therefore, it is better to evaluate the association between As methylation capacity and human diseases with the consideration of potential confounders that might be influential to As methylation. Thus, our study aimed to investigate the possible difference in urinary As metabolites and the methylation capacity between subjects with and without skin lesions by adjustment for potential factors in a highly exposed population of rural China.

## 2. Materials and Methods

### 2.1. Study Area and Subjects

This study was conducted in two villages of Huhhot Basin, which is situated to the northern bank of the Yellow River and to the southern edge of Da Qing Mountains. This region was reported as an endemic arsenism area, where the groundwater is known to be contaminated with naturally geogenic As [21].

According to the Declaration of Helsinki Ethical Principles for Medical Research Involving Human Subjects, a cross-sectional study approved by Ethics Committee of China Medical University was carried out in the two villages. In brief, adult inhabitants (above 18 years old) of Han nationality were included in the study based on the following requirements: (1) residents who lived in one of the two villages for at least 10 years and (2) who had not ingested seafood in the past one week. Pregnant women were excluded. A total of 302 subjects participated in this study. Informed consent was read and signed by all of the participants at the beginning of the survey. A questionnaire was used to obtain information of each individual including sex, age, type of work, socioeconomic conditions, lifestyle of smoking and drinking, dietary habits, and medical history. Medical examination, including measurement of standing height and body weight, was performed by board certified medical doctor according to the standard methods.

### 2.2. Skin Examination

Physical examinations, including a full skin exam, were performed by board certified medical doctor according to the Diagnosis Standards on Endemic Arsenicosis of China (WS/T211-2001, Ministry of Health 2001). In brief, subjects who had obvious skin abnormalities, including hyperpigmentation or depigmentation on trunk and hyperkeratosis on the palms and soles were diagnosed as arsenicosis [22].

### 2.3. Urine Sample Collection and Arsenic Analysis

Spot urine sample was obtained from each participant into a polypropylene container, kept on ice, immediately transferred to the Center for Disease Control in Huhhot, and kept at −20 °C at a refrigerator. These samples were then shipped on dry ice to the Laboratory of Arsenic Analysis at China Medical University (Shenyang, China), stored at −20 °C and finally measured for As species within 3 months.

Determination of urine As metabolites, iAs, MMA, DMA, and trimethylarsonic acid (TMA), was performed by hydride generation-cold trap-atomic absorption spectrophotometer (HG-cold trap-AAS) procedure. This method is reliably and has been commonly used to determine As species in water and biological samples [23,24]. Procedure for detailed quantitation of As species can be found elsewhere [25].The detection limit of this method for those As species is 1 ng. A standard As reference material, SRM 2670 (National Institute of Standards and Technology [NIST], Gaithersburg, MD, USA), containing 480 ± 100 μg/L As was used as a quality control to check the validity of urinary As species measurement. The value determined by our system was 474 ± 20 μg/L. The reliability of four As species was evaluated by the analytical recovery rates of added As species. Spiking urine samples with 10 μg/L of iAs, MMA, DMA and TMA resulted in recoveries of 81%–92%, 88%–98%, 89%–103% and 80%–95%, respectively. Individual urinary creatinine (Cr) was measured by Jaffe reaction as described previously. The final reported urinary As metabolites concentration was adjusted by Cr to remove the influence of urine dilution in spot urine sample. 

### 2.4. Statistical Analysis

As TMA was not detected in any sample, total As (tAs) concentration in urine was reported by summing up the concentrations of iAs, MMA and DMA. The proportions of urinary As metabolites were defined as iAs/tAs × 100% (iAs%), MMA/tAs × 100% (MMA%) and DMA/tAs × 100% (DMA%). Two methylation indices, including PMI [(MMA + DMA)/tAs] and SMI [DMA/(MMA + DMA)], were used to evaluate As methylation capacity. BMI was calculated as weight in kilograms divided by the square of height in meters for all participants. Data with a skewed distribution were logarithmically transformed before statistical tests. The differences of urianry As species and methylation indices between subgroups of sex, age, BMI, smoking and drinking were analyzed by Student’s t-test. The differences of urinary As metabolites and the methylation capacity between subjects with and without skin lesions were compared by Analysis of Covariance (ANCOVA), which is an analysis procedure for looking at group effects on a continuous outcome when some other explanatory variable also has an effect on the outcome. Multivariate-adjusted means were estimated by general linear models with fixed factors and covariates in different models, including sex in Model 1, sex, age and BMI in Model 2, and sex, age, BMI, smoking and drinking in Model 3. These analyses were conducted using the Statistical Package for the Social Sciences (SPSS, version 16.0, SPSS Inc., Chicago, IL, USA). The *p*-value for statistical significance was defined as *p* < 0.05. 

## 3. Results

### 3.1. Baseline Characteristics of the Study Population

The detailed demographic characteristics of the study population have been described in our previous study [25]. In brief, 302 subjects, including 120 males and 182 females, were involved in this study. The proportions of smoker (current or former) and drinker (current or former) were 35.4% (107) and 22.5% (68), respectively. The average age and BMI of the study population were 51 ± 12 years old and 23.9 ± 3.8 kg/m^2^, respectively. In total, 79 subjects were identified to have skin lesions, which characterized by skin pigmentation, depigmentation and/or hyperkerotosis. Urinary As species and methylation indices of the study population were listed in Table 1. The mean concentrations of urinary iAs, MMA, DMA and tAs were 108.3, 84.4, 320.8, and 513.6 μg/g Cr, respectively. DMA was the major As species in urine, followed by iAs and MMA. The average proportion of DMA, iAs and MMA were 60.9%, 23.7% and 15.5%, respectively. Other two As methylation indices, namely PMI and SMI, were 0.76 and 0.80, respectively.

### 3.2. Differences of Urinary Arsenic Species and Methylation Indices among Subgroups

Table 2 presents the differences of urinary As species and methylation indices among subgroups of sex, age, BMI, smoking and drinking. Levels of DMA, tAs, DMA% and SMI were significantly lower in males than in females, while iAs% was significantly higher in males. No sex differences were observed in iAs, MMA, MMA% and PMI. Except iAs, DMA% and SMI, age significantly influenced other As profiles. There was no difference in As metabolites and methylation indices between subjects with higher BMI (>25 kg/m^2^) and lower BMI (≤25 kg/m^2^), as well as smokers and non-smokers. Compared with never drinking subjects, current or former drinking subjects had higher iAs%. However, there were no differences in other As profiles between them.

**Table 1:** Urinary arsenic species and methylation indices of the study population (*n* = 302).

Variables ^a^	Study Population	Study Population	
		Without Skin Lesions (*n* = 223)	With Skin Lesions (*n* = 79)
Arsenic species (μg/g Cr)			
iAs	108.3 ± 138.9	95.9 ± 83.7	143.4 ± 229.9
MMA	84.4 ± 65.1	77.9 ± 65.9	103.0 ± 59.5
DMA	320.8 ± 232.8	314.2 ± 243.0	339.6 ± 201.4
tAs	513.6 ± 362.2	487.9 ± 341.2	586.0 ± 409.7
Arsenic methylation indices			
iAs%	23.7 ± 15.5	23.9 ± 16.6	22.9 ± 11.8
MMA%	15.5 ± 6.7	14.5 ± 6.2	18.2 ± 7.2
DMA%	60.9 ± 13.7	61.6 ± 13.9	58.9 ± 13.1
PMI	0.76 ± 0.15	0.76 ± 0.17	0.77 ± 0.12
SMI	0.80 ± 0.09	0.81 ± 0.07	0.76 ± 0.11

^a^ Values are mean ± SD.

### 3.3. Differences of Urinary Arsenic Species between Subjects with and without Skin Lesions

Crude and adjusted means of the urinary As species in subjects with and without skin lesions are presented in Table 3. The crude values of iAs, MMA and tAs were significantly higher in subjects with skin lesions than those of subjects without skin lesions (*p* < 0.05). The difference of crude value of DMA was not significant (*p* = 0.128). The significant differences in iAs, MMA and tAs were strengthened by adjusting sex in Model 1 and a significant difference of DMA was newly observed between those two groups. After further adjustments for age and BMI (Model 2), the significant differences in iAs and MMA were attenuated, while differences in DMA and tAs observed in Model 1 were disappeared. Further adjustments of Model 2 for smoking and drinking, the significant differences in iAs and MMA were retained, while the differences in DMA and tAs were also disappeared.

### 3.4. Differences of Urinary Arsenic Methylation Indices between Subjects with and without Skin Lesions

Table 4 shows the differences of crude and adjusted means of the urinary As methylation indices between subjects with and without skin lesions. The crude values of MMA% was significantly higher in subjects with skin lesions than those of subjects without skin lesions, while SMI was significantly lower in subjects with skin lesions. After adjusting sex in Model 1, the significant difference in MMA% and SMI were attenuated (*p* = 0.002 and 0.006, respectively). The significant differences in MMA% and SMI were attenuated by further adjustments for age and BMI (Model 2). Further adjustments of Model 2 for smoking and drinking, the significant differences in MMA% and SMI were little changed. No differences were observed in iAs%, DMA% and PMI between subjects with and without skin lesions before and after the adjustments.

**Table 2:** Differences of urinary arsenic species and methylation indices between subgroups.

Variables	*n*	iAs (μg/g Cr) ^a^	MMA (μg/g Cr) ^a^	DMA (μg/g Cr) ^a^	tAs (μg/g Cr) ^a^	iAs% ^a^	MMA% ^a^	DMA% ^a^	PMI ^a^	SMI ^a^
Sex										
Male	120	98.4 ± 80.8	80.6 ± 58.1	266.2 ± 191.7	445.2 ± 277.1	25.5 ± 15.5	16.8 ± 7.6	57.7 ± 14.0	0.75 ± 0.15	0.78 ± 0.11
Female	182	114.9 ± 166.4	87.0 ± 69.4	356.8 ± 250.3	558.7 ± 403.2	22.5 ± 15.4	14.6 ± 5.8	63.0 ± 13.1	0.78 ± 0.15	0.81 ± 0.07
*p*-value		0.425	0.578	0.006	0.029	0.042	0.183	0.039	0.455	0.039
Age (years) ^b^										
≤51	155	118.8 ± 180.8	72.4 ± 62.5	277.5 ± 210.8	468.7 ± 376.5	27.2 ± 17.7	14.3 ± 6.6	58.5 ± 14.5	0.73 ± 0.18	0.81 ± 0.08
>51	147	97.3 ± 71.3	97.2 ± 65.7	366.5 ± 246.4	560.9 ± 341.5	19.9 ± 11.7	16.7 ± 6.5	63.5 ± 12.3	0.80 ± 0.12	0.79 ± 0.10
*p*-value		0.657	<0.001	0.003	0.010	<0.001	0.001	0.054	<0.001	0.145
BMI (kg/m^2^)										
≤25	213	105.1 ± 86.2	86.1 ± 65.4	312.2 ± 210.6	503.4 ± 312.6	23.6 ± 14.9	16.0 ± 6.8	60.5 ± 13.3	0.76 ± 0.15	0.79 ± 0.09
>25	89	116.1 ± 219.2	80.4 ± 64.7	341.5 ± 278.9	538.0 ± 461.1	23.8 ± 16.9	14.3 ± 6.3	61.9 ± 14.5	0.76 ± 0.17	0.82 ± 0.07
*p*-value		0.144	0.199	0.748	0.451	0.323	0.125	0.415	0.980	0.171
Smoking										
Current or former	107	96.5 ± 76.1	85.3 ± 64.4	296.8 ± 200.5	478.6 ± 295.3	23.0 ± 14.7	16.3 ± 6.6	60.7 ± 12.8	0.77 ± 0.15	0.79 ± 0.08
Never	195	114.8 ± 163.3	84.0 ± 65.7	334.0 ± 248.2	532.7 ± 393.6	24.0 ± 15.9	15.0 ± 6.7	61.0 ± 14.2	0.76 ± 0.16	0.80 ± 0.09
*p*-value		0.466	0.742	0.777	0.575	0.823	0.253	0.671	0.573	0.957
Drinking										
Current or former	68	105.0 ± 88.0	78.7 ± 65.3	258.7 ± 177.6	442.4 ± 274.0	27.8 ± 17.8	15.9 ± 7.0	56.3 ± 14.6	0.72 ± 0.18	0.78 ± 0.08
Never	234	109.3 ± 150.7	86.1 ± 65.1	338.9 ± 243.9	534.3 ± 382.1	22.5 ± 14.6	15.3 ± 6.6	62.2 ± 13.1	0.78 ± 0.15	0.80 ± 0.09
*p*-value		0.475	0.500	0.102	0.255	0.008	0.968	0.112	0.082	0.755

^a^ Values are mean ± SD; ^b^ Cut point is median value of this variable.

**Table 3:** Differences of adjusted means of urinary arsenic species between subjects with and without skin lesions.

Characteristics	Study Population		*p*-value
	Without Skin Lesions (*n* = 223)	With Skin Lesions (*n* = 79)	
iAs (μg/g Cr) ^a^			
Crude ^b^	69.7 (62.4, 78.0)	99.3 (78.7, 114.6)	0.006
Model 1 ^c^	68.1 (60.7, 76.4)	95.9 (79.4, 115.6)	0.003
Model 2 ^d^	68.2 (60.7, 76.7)	96.4 (79.1, 117.5)	0.005
Model 3 ^e^	70.6 (61.8, 80.9)	99.1 (80.5, 121.9)	0.006
MMA (μg/g Cr) ^a^			
Crude ^b^	41.7 (34.7, 50.1)	79.1 (58.1, 107.7)	0.001
Model 1 ^c^	40.2 (33.2, 48.6)	80.2 (58.9, 109.4)	<0.001
Model 2 ^d^	42.5 (35.0, 51.5)	68.7 (49.8, 95.06)	0.015
Model 3 ^e^	42.9 (34.4, 53.5)	69.3 (49.4, 97.3)	0.016
DMA (μg/g Cr) ^a^			
Crude ^b^	208.9 (182.0, 239.9)	257.0 (204.2, 324.3)	0.128
Model 1 ^c^	195.9 (170.2, 225.4)	264.2 (210.4, 331.9)	0.030
Model 2 ^d^	202.3 (175.4, 233.4)	238.8 (187.9, 303.4)	0.261
Model 3 ^e^	206.1 (175.0, 242.7)	243.8 (189.7, 313.3)	0.255
tAs (μg/g Cr) ^a^			
Crude ^b^	355.6 (317.7, 399.0)	466.7 (385.5, 564.9)	0.017
Model 1 ^c^	339.6 (302.0, 381.9)	476.4 (393.6, 575.4)	0.003
Model 2 ^d^	348.3 (309.0, 392.6)	440.6 (361.4, 538.3)	0.054
Model 3 ^e^	354.0 (309.0, 406.4)	447.7 (363.1, 552.1)	0.057

^a^ Values are adjusted mean and 95% confidence interval (95% CI); ^b^ Crude model; ^c^ Model 1 adjusted for sex. ^d^ Model 2 adjusted for variable in Model 1, age and BMI; ^e^ Model 3 adjusted for variables in Model 2, smoking and drinking.

**Table 4:** Differences of adjusted means of urinary arsenic methylation indices between subjects with and without skin lesions.

Characteristics	Study Population		*p*-value
	Without Skin Lesions (*n* = 223)	With Skin Lesions (*n* = 79)	
iAs% ^a^			
Crude ^b^	19.6 (18.1, 21.2)	20.4 (17.8, 23.3)	0.632
Model 1 ^c^	20.0 (18.5, 21.8)	20.1 (17.6, 23.0)	0.955
Model 2 ^d^	19.6 (18.0, 21.2)	21.9 (19.1, 25.1)	0.190
Model 3 ^e^	20.0 (18.2, 21.9)	22.1 (19.2, 25.6)	0.216
MMA% ^a^			
Crude ^b^	11.7 (10.5, 13.1)	16.9 (14.1, 20.3)	0.001
Model 1 ^c^	11.9 (10.6, 13.3)	16.9 (14.1, 20.2)	0.002
Model 2 ^d^	12.2 (10.9, 13.7)	15.6 (12.9, 18.9)	0.037
Model 3 ^e^	12.1 (10.6, 13.8)	15.5 (12.7, 16.9)	0.036
DMA% ^a^			
Crude ^b^	58.8 (55.3, 62.4)	55.2 (49.9, 61.0)	0.294
Model 1 ^c^	57.7 (54.3, 61.4)	55.6 (50.2, 61.4)	0.525
Model 2 ^d^	58.1 (54.6, 61.9)	54.2 (48.8, 60.1)	0.273
Model 3 ^e^	58.2 (54.2, 62.5)	54.5 (48.8, 60.8)	0.304
PMI ^a^			
Crude ^b^	0.72 (0.69, 0.76)	0.76 (0.70, 0.82)	0.322
Model 1 ^c^	0.72 (0.68, 0.76)	0.76 (0.70, 0.83)	0.241
Model 2 ^d^	0.73 (0.69, 0.77)	0.74 (0.68, 0.80)	0.803
Model 3 ^e^	0.72 (0.68, 0.76)	0.73 (0.67, 0.80)	0.754
SMI ^a^			
Crude ^b^	0.81 (0.78, 0.84)	0.73 (0.69, 0.77)	0.001
Model 1 ^c^	0.80 (0.77, 0.83)	0.73 (0.69, 0.77)	0.006
Model 2 ^d^	0.80 (0.77, 0.83)	0.73 (0.69, 0.78)	0.024
Model 3 ^e^	0.81 (0.78, 0.84)	0.74 (0.70, 0.79)	0.025

^a^ Values are adjusted mean and 95% confidence interval (95% CI); ^b^ Crude model; ^c^ Model 1 adjusted for sex; ^d^ Model 2 adjusted for variable in Model 1, age and BMI; ^e^ Model 3 adjusted for variables in Model 2, smoking and drinking.

## 4. Discussion

In this study, we investigated urinary As metabolites and the methylation capacities between subjects with and without skin lesions in Inner Mongolia. We found that demographic characteristics such as sex, age, and drinking can affect As metabolism. The absolute values of iAs and MMA were significantly higher in subjects with skin lesions than in controls before and after adjustment for several covariates. MMA% was significantly higher in subjects with skin lesions, whereas SMI was significantly higher in subjects without skin lesions. Although an adjustment for potential confounding variables attenuated the significant differences, the differences in MMA% and SMI did not change much between the two groups. Our findings of higher iAs, MMA and MMA% and lower SMI values in skin lesions group indicate that subjects with higher levels of more toxic metabolites and lower methylation capacity may prone to As-induced skin lesions, although the cross-sectional study can only provide weak evidence of causality.

Inorganic As from drinking water is the primary source of human exposure. After metabolizing in liver, As is excreted in the urine primarily through the kidneys in a mixture of inorganic, monomethylated, and dimethylated forms [26]. Urinary As concentration is a good biomarker for internal dose and is considered as the most reliable indicator of recent exposure to iAs [1]. Previous studies have indicated that the relative distribution of iAs, MMA and DMA in urine of most population groups seem to be constant and have on average of 10%–30%, 10%–20% and 60%–70%, respectively [27]. In this study, high levels of As in urine indicated that inhabitants of those two villages are at risk of high As exposure. Our results showed that the average percentages of As metabolites (iAs, MMA and DMA) in urine were 23.66%, 15.46%, and 60.89%, respectively, which consistent with previously studies (Table 1). However, there are some exceptions. Indigenous people in northern Argentina excrete only a small percentage of MMA in urine [28,29], whereas people in Taiwan excrete 20%–30% on average of MMA in urine [30]. Although the ethnic difference in the distribution of urinary As metabolites is still unclear, recent studies have indicated that genetic polymorphisms may be responsible for a large part of the variation of As metabolism between different population groups [6,31].

In this study, urinary As metabolites were compared between different subgroups including sex, age, BMI, smoking and drinking (Table 2). Women had a greater urinary excretion of tAs and DMA than those of men. Our results showed that the percentages of urinary As metabolites varied widely between men and women. Men had a lower As methylation capacity than women, which is characterized by significantly lower DMA% and SMI values and higher iAs% value. This finding was in accordance with many other studies, which suggested that women had better As methylation capacity than that of men [32,33]. The sexual difference in As methylation capacity can be partly explained by the effect of estrogen, which can increase the synthesis of choline by up-regulate phosphatidylethanolamine methyltransferase [34,35]. The choline was then oxidized into betaine, which can donate its methyl group for the regeneration of methionine from homocysteine [36]. Efficient one-carbon metabolism required adequate supply of methyl group donors can facilitate As methylation and elimination [37]. Therefore, it is reasonable to observe a better methylation capacity in women. 

In this study, adult inhabitants older than 18 years were recruited, since most epidemiologic studies have agreed with a better methylation capacity in children [24,38]. Our results showed that older people had higher As excretion in urine than younger people. Except DMA% and SMI, age significantly influenced other percentages of As metabolites (iAs% and MMA%) and the methylation index (PMI). This indicated that age may play a role in As metabolism. Studies in Taiwan have suggested that increasing MMA% and decreasing DMA% and SMI were associated with increasing age [39,40]. Similarly in a recent study carried out in Argentina, although there were no statistically significant difference in MMA%, DMA% and SMI among different age groups, aging was associated with a significantly decreasing iAs% in urine [41]. In our study, the lower iAs% as well as higher MMA% and PMI in older group indicated that As methylation is incomplete among older persons, especially the second methylation capacity. Although the age effect on the As methylation capacity needs to be further confirmed, it was most likely explained by some confounding factors. Firstly, age is a possible surrogate for the duration of residence, which is an indicator of cumulative As exposure. Secondly, older age is probably associated with various functional changes in the organs involved in As metabolism. 

BMI is a measure for indicating obesity or nutritional status, which may have an effect on As metabolism. Studies in Taiwan revealed that BMI was positively associated with DMA% and negatively associated with MMA% and tAs [40,42]. In the study by evaluating the association between BMI and As methylation efficiency, higher BMI was found to be associated with low MMA% or high SMI [43]. However, in this study, BMI was found to be not associated with any of the urinary As species or methylation indices. The effect of smoking on As metabolism is inconsistent. Some studies suggested that smoking is associated with a poorer methylation capacity [19,39]. However, some other studies suggested that smoking is not associated with As methylation capacity [41]. Our results showed that smoking was not associated with any of the urinary As metabolites or methylation indices. Additionally, we found that non-drinkers have lower iAs% than that of drinkers. This is in accordance with the findings of Hopenhayn-Rich *et al.* [44], which suggested that alcohol drinkers had a higher iAs%. However, the alcohol effects on As methylation cannot be confirmed in studies performed in Taiwan [39]. Therefore, more research works are required to provide evidence that drinking could exert an effect on As metabolism. 

The mechanisms of As toxicity are not fully elucidated. Methylation of iAs has been previously thought to be a detoxification pathway. However, recent studies have confirmed that trivalent intermediates, such as MMA^III^ and DMA^III^, are produced during As metabolism and are more toxic than other metabolites [45,46]. It has been demonstrated that urinary As profile is correlated well for plenty of chronic effects related to As concentrations in drinking water [7]. Analyses of the urinary As profile can give a hint to the methylation capacity of exposed individuals. In this study, no significant difference were found in tAs excretion between subjects with and without skin lesions, which indicating similar As exposure among the study population. Although urinary DMA were similar in the two groups, we found that subjects with skin lesions had a higher urinary iAs and MMA than subjects without skin lesions by adjustment for several potential confounders (Table 3). Our results suggested that an individual susceptible to As-induced skin lesions may excrete more toxic metabolites than the controls under the same As exposure level. It was shown that cases with skin lesions had a higher urinary MMA% and lower SMI than controls by adjustment for sex, age, BMI, smoking and drinking (Table 4). This indicated that cases with skin lesions have a lower methylation capacity, especially the secondary methylation step. Several studies conducted in Taiwan, Mexico and Bangladesh have found that higher percentage of MMA was associated with a higher risk of skin lesions [13,15,47,48]. In this study, we were unable to determine MMA^III^ directly. Therefore, urinary MMA was used to be a marker of MMA^III^ in human body. Although the degree to which urinary MMA may be an accurate surrogate for MMA^III^ level is unclear, the confirmed highly toxic of MMA^III^ supports our findings that higher MMA is associated with As-induced skin lesions.

## 5. Conclusions

In summary, the present study suggested that As metabolism can be influenced by demographic characteristics such as sex, age, BMI and drinking. Subjects with skin lesions had a lower As methylation capability than subjects without skin lesions, which characterized by higher urinary excretion of toxic iAs and MMA, as well as higher MMA% and lower SMI values.
